# Rao-Blackwellized Particle Filter Algorithm Integrated with Neural Network Sensor Model Using Laser Distance Sensor

**DOI:** 10.3390/mi14030560

**Published:** 2023-02-27

**Authors:** Amirul Jamaludin, Norhidayah Mohamad Yatim, Zarina Mohd Noh, Norlida Buniyamin

**Affiliations:** 1 Centre for Telecommunication Research & Innovation (CeTRI), Fakulti Kejuruteraan Elektronik & Kejuruteraan Komputer (FKEKK), Universiti Teknikal Malaysia Melaka (UTeM), Durian Tunggal 76100, Melaka, Malaysia; 2 Faculty of Electrical Engineering, Universiti Teknologi MARA, Shah Alam 40450, Selangor, Malaysia

**Keywords:** SLAM, occupancy grid map, artificial neural network, laser distance sensor, particle filter

## Abstract

Commonly, simultaneous localization and mapping (SLAM) algorithm is developed using high-end sensors. Alternatively, some researchers use low-end sensors due to the lower cost of the robot. However, the low-end sensor produces noisy sensor measurements that can affect the SLAM algorithm, which is prone to error. Therefore, in this paper, a SLAM algorithm, which is a Rao-Blackwellized particle filter (RBPF) integrated with artificial neural networks (ANN) sensor model, is introduced to improve the measurement accuracy of a low-end laser distance sensor (LDS) and subsequently improve the performance of SLAM. The RBPF integrated with the ANN sensor model is experimented with by using the Turtlebot3 mobile robot in simulation and real-world experiments. The experiment is validated by comparing the occupancy grid maps estimated by RBPF integrated with the ANN sensor model and RBPF without ANN. Both the results in simulation and real-world experiments show that the SLAM performance of RBPF integrated with the ANN sensor model is better than the RBPF without ANN. In the real-world experiment results, the performance of the occupied cells integrated with the ANN sensor model is increased by 107.59%. In conclusion, the SLAM algorithm integrated with the ANN sensor model is able to improve the accuracy of the map estimate for mobile robots using low-end LDS sensors.

## 1. Introduction

Mobile robotics is one of the most recent research topics that has gained popularity and interest in either academic or commercial research. In mobile robotics applications, there are two main tasks that have to be solved for a robot to learn the environment, which are localization and mapping the environment [1]. This task is called simultaneous localization and mapping (SLAM). Most mobile robot platforms use high-end sensors to solve the SLAM problem, but it requires a high cost to develop the robot. Alternatively, a mobile robot can be equipped with a low-end sensor to reduce the cost of the robot. However, typically low-end sensors have low accuracy. Thus, it introduces challenges to producing a good-quality map of the environment. This issue can be mitigated by integrating the SLAM algorithm with an artificial neural network (ANN) while maintaining the use of low-cost sensors [2,3]. The ANN learner is trained using a noisy dataset from the mobile robot. Then, the model of the ANN is integrated with the SLAM algorithm, which is the Rao-Blackwellized particle filter (RBPF), to perform the SLAM task.

**Figure 1:**
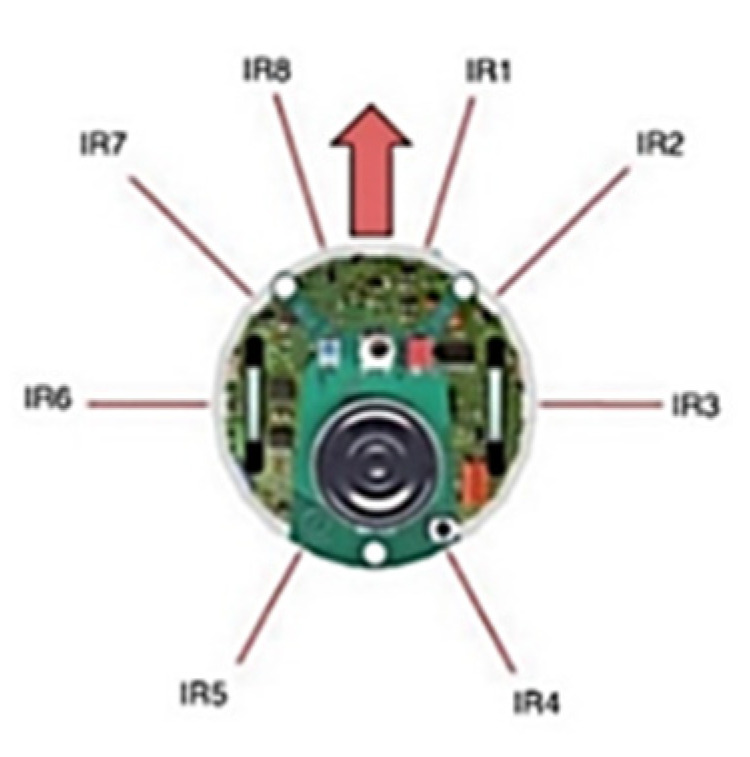
E-puck mobile robot with eight infrared sensors [2]. Reproduced with permission from Norhidayah Mohamad Yatim, Lecture Notes in Electrical Engineering, vol 398., published by Springer, 2017.

**Figure 2:**
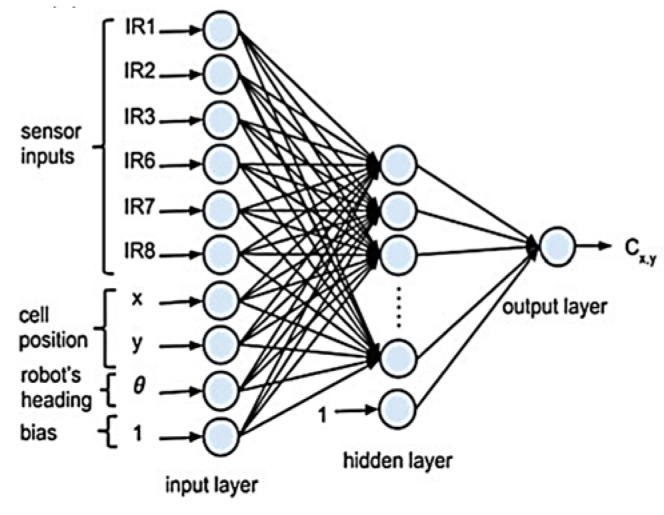
Input of ANN consisting of infrared sensors measurements, cell position, and heading of the robot [2]. Reproduced with permission from Norhidayah Mohamad Yatim, Lecture Notes in Electrical Engineering, volume 398, published by Springer, 2017.

The characteristics of the sensor influence the accuracy of the map, particularly the accuracy of the obstacle sensing location [4]. Using high-cost sensors such as SICK LIDAR and Hokuyu LIDAR can achieve high accuracy in map estimation [5,6,7]; using low-cost sensors can result in inadequate map quality [8,9,10,11,12]. As a result, ANN is proposed to be used in this project. ANN is typically composed of an input layer, a hidden layer(s), and a single linear output layer. In [2], the range readings from the front six infrared proximity sensors of the E-puck mobile robot, as shown in Figure 1, were collected to train the ANN model. Then, the range readings, the x and y cell positions of the occupancy grid map (OGM) and the robot’s heading θ are used as the input layer. The occupancy value of each cell is used as the output layer of the training, as shown in the ANN configuration in Figure 2 [2]. A similar architecture of ANN is used in [3,13]. In [13], they used all eight infrared sensors of the Khepera robot, which include the cell positions around the robot in the range of the sensors as the ANN input. If the sensor’s range is up to 0.25 m, as in the case of the Khepera infrared sensor, then the cells included are only within that range. In [3], the author used the front four sensor measurements of the Khepera III robot, which also includes the cell positions. The cell positions are encoded using the distance, *d,* and angle, θ, to the closest sensor rather than Cartesian coordinate as [2,13]. The output of the network is the occupancy probability value of the cell that would incrementally build the OGM.

OGM consists of cells, where each cell is a small rectangular part of the world with equal size [14]. The cell is modeled on a binary random variable showing the probability of the existence of an object in the cell where any cell in the grid has a probability value showing whether there is free space or part of an obstacle in the corresponding region. The occupancy value of a cell depends on the distance determined when the coincident cells (i.e., black cells) are considered occupied, and the cells between them (i.e., white cells) are called free cells [15]. The grey cells are an unknown region that has not been explored yet. The grid map, also known as the location map, displays the cell matrix environment where the cell uses x and y positions. OGM is the best way to represent the real-world structure, similar to a blueprint of the environment in a 2D map [16].

In [4], there is only single input which is the reading of sensor measurement from the infrared range-finder PB9-01 and the actual distance from the sensor to obstacles is the target output of ANN training. For this method, ANN is used to train the noisy sensor measurement from the infrared range-finder PB9-01 in order to minimize the sensor error. The ANN generates its own output and then compares it to the target output. If the network output varies from the target output, which is the actual distance from the sensor to obstacles, weights between the nodes are adjusted to minimize the discrepancies using a training rule.

In the context of computation time, estimation of ANN by using distance to the obstacles is considered faster [4] than estimation by using each of the grid cells [2,3,13]. This is due to the estimation by distance does not evaluate each cell, only those at the endpoint of the sensor reading. In [2,3,13], the estimation of the map computes each cell of the region detected with an occupancy value ranging from 0 to 1. Some of the cells may not have a value of exactly 0 or 1 due to the uncertainty in cell occupancy. Cell-by-cell evaluation can cause slow computation time. Hence, real-time implementation is not feasible. 

In the second method [4], an ANN is used to obtain the corrected distance measurement to the barrier. After that, this measurement is used to build the grid map for the detected region. In this region, only two values are considered, which are value 0 for the free space and 1 for the occupied space (i.e., obstacles in the space) to compute the grid map estimation. Hence, ANN computation is not required for each grid cell but for the entire detected region. This technique is considered easier and faster compared to cell-by-cell estimation. Therefore, real-time implementation is more suitable with this method.

To solve the SLAM problem, a SLAM algorithm is required in this project. Some of the examples of the algorithm used in SLAM are the extended Kalman filter (EKF), particle filter (PF), and graph SLAM. Kalman filter is a linear recursive state estimator [17,18]. Extended KF (EKF) is a nonlinear version of the Kalman filter, and it implements the multivariate Taylor series expansions in order to linearize the nonlinear model [19]. EKF is based on Gaussian distributions, and the problem with the nonlinear model leads to non-Gaussian distributions. Thus, applying the standard Kalman filter to nonlinear systems with additive Gaussian noise is achieved by continually updating a linearization on the previous state estimate, starting with an initial guess. In EKF, the computational complexity of the correctional stage increases quadratically with the number of points of reference. This is a challenge in real-world practice. The linearization of the motion model and observation models in EKF also may produce unconvincing results [20]. This is especially noticeable when the systems are extremely nonlinear, and it might cause the filter to deviate.

Particle filter is a powerful method and is used in many applications, including filtering, tracking, and navigation, with an extremely nonlinear method and a very wide state space [21,22,23]. A particle filter is a Bayes filter approximation that represents the robot pose via an arbitrary multimodal distribution of probability using a set of particles [24]. It uses random sampling instead of a closed form to represent the estimated belief, while each particle contains a map of the environment model [25]. This method is computationally costly since the algorithm requires large samples of particles in order to obtain adequate results [26]. However, it is able to address the issue that EKF cannot solve, such as non-Gaussian noise or extremely nonlinear systems. Unlike EKF, particle filters can estimate the map of the environment without the need for landmarks or feature detection and can process raw data from sensor measurements [14]. Due to this, the particle filter is suitable for occupancy grid map (OGM) representation, which uses raw data sensor measurements to build the map. In contrast, the EKF algorithm is more suitable for feature maps because of the nature of the algorithm uses landmarks or features in map estimation [25]. Rao-Blackwellized particle filters (RBPF) is a version of particle filter-based SLAM that is an effective implementation of particle filter-based SLAM. By using Gaussian substructures in the model, RBPF improves the efficiency of the algorithm [25,27]. In this approach, RBPF approximates the distribution of the robot’s pose belief while each particle carries an individual map of the environment. The main contribution of this method is reducing the number of particles used [27]. This method takes only the accurate proposal distribution and the robot movement, plus the most recent observation. This can drastically decrease the uncertainty of the robot’s poses in the prediction step of the filter. RBPF also can address the loop detection problem. This is due to the algorithm having a good local continuity by using particle distribution [28,29]. In addition, it has computational cost flexibility by adjusting the number of particles. The computational cost decreases as the number of particles decreases. [14,23]. By using a lower number of particles, the experiment can be conducted efficiently with respect to the specifications of the computer. Therefore, the RBPF algorithm integrated with ANN is implemented in this paper and observed the difference after the ANN implementation.

The main contribution of this paper can be summarized as:

- The improvement of the measurement accuracy of a low-end laser distance sensor (LDS) using ANN.
- The improvement of the performance of the SLAM algorithm by integrating the RBPF algorithm with the ANN sensor model.

The organization of this paper is as follows: Section 2 describes the methodology of the ANN sensor model training and RBPF algorithm framework integrated with the ANN sensor model. Section 3 analyzes the performance of the ANN sensor model after the training and reports the results of the RBPF algorithm after integrating with the ANN sensor model and before integrating with ANN. Lastly, Section 4 concludes the finding of this paper.

## 2. Methodology

To implement the SLAM algorithm integrated with the ANN sensor model, the methodology is divided into four phases. Firstly, the data collection of LDS sensor measurement occurs, which is the distance between the sensor and the wall. Then, if the data collection is satisfactory, the data (input to the ANN model) are mapped with the reference data, which is the real distance between the sensor and the wall (output of the ANN model) to train the ANN model. In the third phase, the data are trained until a sufficient model of ANN is obtained. Then, the obtained ANN model is implemented and integrated with the SLAM algorithm. In the last phase, the performance of the SLAM algorithm integrated with the ANN sensor model is evaluated by conducting simulation and real-world experiments using the Turtlebot3 mobile robot platform. The performance is evaluated by using a map score for the evaluation of the map’s accuracy.

### 2.1. Data Collection

Feed-forward backpropagation ANN requires inputs to train the ANN model. For this purpose, sensor measurements from the LDS-01 sensor are collected as the input of the ANN. There are two datasets collected, which are data for the simulation model and data for the real-world model. 

**Figure 3:**
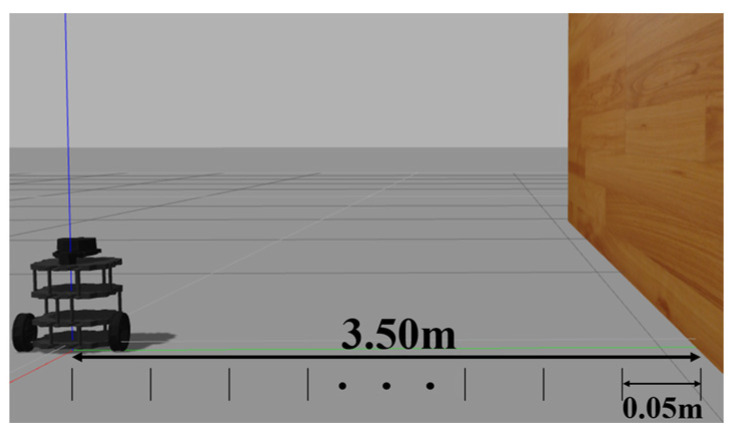
Turtlebot3 position ranging from 0.05 m to 3.5 m to the wall.

**Figure 4:**
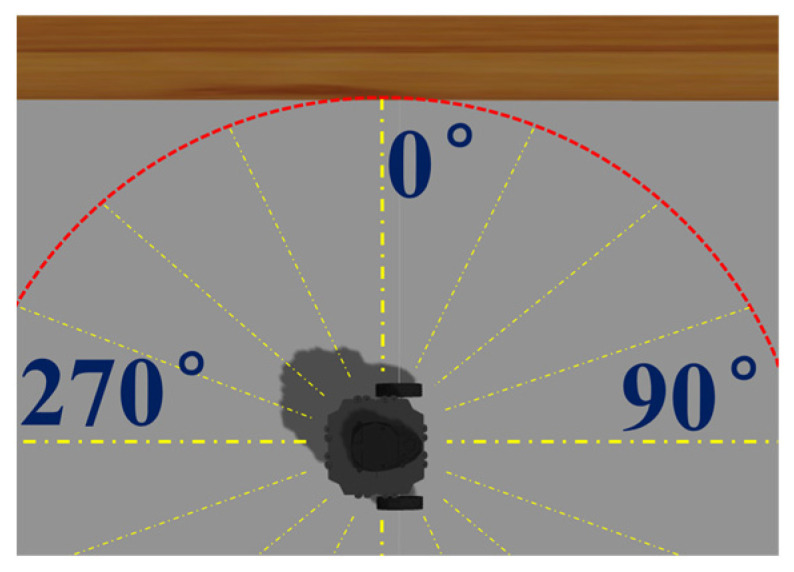
Turtlebot3 position at 0° perpendicular to the wall.

For the simulation, the sensor measurement (i.e., input to ANN simulation model) between the mobile robot and the wall in the range of 0.05 m to 3.5 m was taken, as shown in Figure 3. In each range, about 2500 data are collected as the input of the ANN. The measurements are taken only at a 0° angle. This is because, during the data collection phase, the robot is placed such that the sensor measurement at 0° is perpendicular to the wall, as shown in Figure 4. In the real-world experiment, the sensor measurement between the mobile robot and the wall in the range of 0.1 m to 3.5 m is collected. For every space of 0.1 m, 2000 data were collected. Then, these data are trained to build an ANN model for a real-world robot platform.

### 2.2. Training the ANN Sensor Model

ANNs are beneficial for nonlinear modeling because of their ability to approximate any arbitrary function. In this study, the system employs a multilayer feed-forward network. The network is made up of an input layer, a hidden layer, and a single linear layer for output.

In machine learning, using the ANN method is commonly classified as supervised learning. The method of training is so named because the ANN regressor must learn how each training input vector sample is associated with a related label named the target output [2]. The ANN comprises a neuron topological graph that computes the input activation function of each neuron and sends the result to the output layer. Suppose $x_{i}$ denotes a series of input features (LDS sensor data), so the first step in ANN is to transform the weights and shift inputs by a bias factor fitting for each $a_{j}$ neuron:

(1) $$a_{j}=x_{i}W_{j}^{\left(1\right)}+b_{1}$$

where the $W_{j}^{\left(1\right)}$ are the weights and $b_{1}$ are the bias that assigned to *i*th input and *j*th neuron of the hidden layer. After that, $a_{j}$ is transformed with the selected activation feature, such as sigmoid or tan-sigmoid. A tan-sigmoid activation function given by $z_{j}$ was used in this paper.

(2) $$z_{j}=h\left(a_{j}\right)=\frac{2}{1+e^{-2a_{j}}}-1$$

The output (or target) vector y elements are then computed as:

(3) $$y_{i}=a_{k}=\sum_{j=1}^{M}z_{j}W_{j}^{\left(2\right)}+b_{2}$$

where the $W_{j}^{\left(2\right)}$ are the weights and $b_{2}$ is the bias that assigned to *i*th input (from input layer) and *j*th neuron of the hidden layer. M is the number of neurons of the hidden layer which is the input for the output layer.

It was shown that any continuous function can be correctly approximated using ANN topologies with the right set of weights and biases. The use of an ANN has two phases: a training phase and a test phase. The ANN is trained to return a certain output due to a particular input. During the training phase, the ANN is trained by adjusting the parameters within each layer. The challenge with learning is finding the optimal weights *W* combination to ensure the output of the network is as close as possible to the target output value as possible. The training algorithm attempts to minimize a mean-square-error (MSE) between the target, t and the predicted output value y given by:

(4) $$MSE=\frac{1}{n}\sum_{i=1}^{n}{\left(y_{i}-t_{i}\right)}^{2}$$

The ANN returns the output in the test phase based on the input propagation over all layers. The LIDAR data point from the LDS sensor were represented by the nodes in the input layer, while the target distance was represented by one node in the output layer. The training data series is applied to ANN in order to train the network and to reduce the mean square error function. The most popular method of minimizing error is to use a backpropagation algorithm. Levenberg Marquardt (LM) algorithm is the extension of the backpropagation algorithm, appears to be the quickest approach for training moderate-sized feed-forward neural networks [30,31] and is used in this paper. The output of the network is computed using different numbers of neurons in the hidden layer, ranging from 5 to 60. The number of neurons in the hidden layer is chosen depending on the response of an unseen test set that resulted in the smallest average error using a fixed training set. In addition, the complete data set was split into a training and a validation set to prevent overfitting.

### 2.3. SLAM Algorithm Integrated with ANN Sensor Model

**Figure 5:**
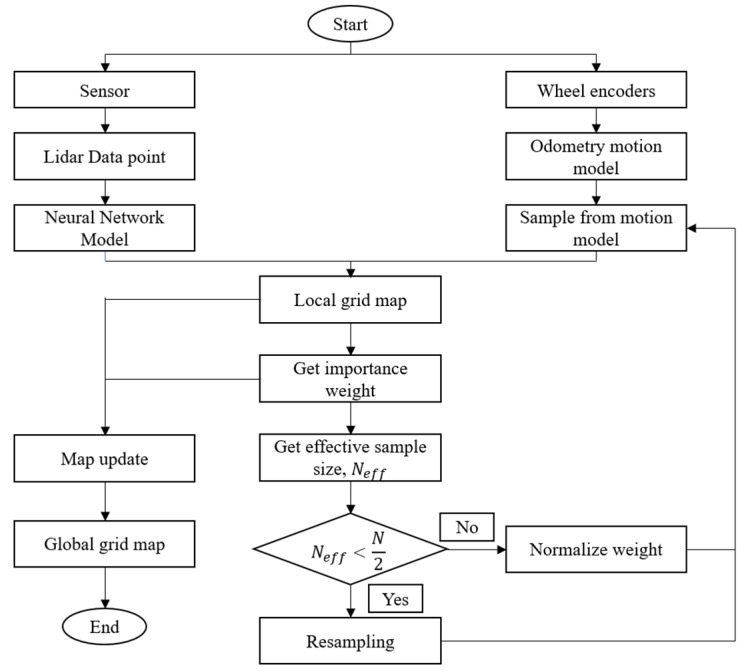
RBPF algorithm integrated with ANN.

The SLAM algorithm that is used in this paper is the Gmapping package that utilizes the Rao-Blackwellized Particle Filters (RBPF) algorithm [32,33,34]. In this experiment, the number of particles used was only 30 particles. Figure 5 shows the Rao-Blackwellized particle filter (RBPF) algorithm integrated with the ANN sensor model that is implemented in this research.

After obtaining the model of the ANN from Section 2.2, the sensor with the ANN model is integrated with the RBPF algorithm, as shown in Figure 5. LIDAR data point from the LDS-01 sensor is used as the input of the ANN model. The LIDAR data point is computed by the ANN model to produce new data that is more accurate. At the same time, odometry or localization-by-odometry is obtained by using the encoders placed on each wheel to calculate the position of the robot [35]. Then, the RBPF algorithm uses the ANN LIDAR data point and odometry motion model that have been obtained to estimate the map environment and the robot’s pose within the map. It updates the collection of samples representing the posterior of the map and the vehicle’s trajectory in four steps:

1. Sampling: A proposal distribution, π is used to sample next-generation particles at time t, $x_{t}^{i}$ from the previous set of weighted particles $x_{t-1}^{i}$. A common practice is to use odometry motion model distribution to approximate the proposal distribution, π.
  (5) $$p\left(x_{t}|x_{t-1},u_{t}\right)$$
2. Importance weighting: Calculate the importance weight, $w_{t}^{i}$, by using the difference between actual observation and predicted observation in (6):
  (6) $$p\left(z_{t}|x_{t}^{i},m_{t-1}^{i}\right)$$

This term is also called measurement likelihood. Here, predicted observation is calculated using the previous global map, $m_{t-1}^{i}$. Due to the implementation of a high variance sensor, the measurement likelihood adapted uses the map matching method [25].

3. Resampling: Particles are resampled according to their weight. Particles with higher weight are the most likely to be resampled for the next generation. All particles have the same weight after resampling. A selective resampling phase is suggested in which the so-called effective number of $N_{eff}$ particles is described as:
  (7) $$N_{eff}=\frac{1}{\sum_{i=1}^{N}{\left(\omega^{i}\right)}^{2}}$$

$N_{eff}$ can be considered as a calculation of the dispersion of the importance weights and the efficiency of the particle set approximation of the true posterior can be pronounced via the formula above. The technique suggested is to resample the N/2 particle number when $N_{eff}$ is smaller than any threshold. The probability of loss of usable particles is minimized considerably since re-sampling takes place only as necessary, and the cumulative number of those operations is decreased [36]. If it is higher than the threshold, the particles will be normalized.

4. Map update: Update the particles’ map estimate conditioned on the robot’s state and current observation by using:
  (8) $$p\left(m_{t}^{i}|x_{1:t}^{i},z_{1:t}\right)$$

For each particle, the corresponding map estimate $p\left(m_{t}^{i}|x_{1:t}^{i},z_{1:t}\right)$ is computed based on the trajectory $x_{1:t}^{i}$ of the particle and the history of observations $z_{1:t}$. In the Gmapping package, the RBPF algorithm is implemented to estimate both the map and the robot’s state.

### 2.4. Evaluation Method of the SLAM Algorithm Integrated with ANN Sensor Model

To investigate the performance of the SLAM algorithm integrated with the ANN sensor model that has been described in Section 2.3, two sets of experiments were designed. The first one is to evaluate the performance of SLAM algorithms without ANN. The second one is to evaluate the performance of SLAM algorithms integrated with the ANN sensor model. Then, both of the algorithms are compared with the ground truth map. The ground truth map is obtained by using the RBPF algorithm, in which the robot explored the environment in more loops than the resulting maps in which the robot explored only one loop. The process is repeated until a satisfactory ground truth map is obtained. By exploring in more loops, the uncertainty in the pose of the robot typically decreases. This is due to the robot’s ability to localize itself in the map that has been built [29]. Due to the uncertainty decreases, the accumulated error of the robot’s state estimate is observed to still be within the bounds and has not diverged. This method is used to obtain the ground truth map in both the simulation and real-world experiments.

A standard software system, which is the robot operating system (ROS Kinetic), is used in this paper in order to efficiently conduct the robot experiments. ROS can minimize the time it takes to prepare a solution for testing. This is due to the ROS having available software libraries and tools such as Gazebo simulation, ROS Visualizer (RViz), and rosbag that can help to build and test the robot applications. Furthermore, it has a software framework that makes it easy for users to develop modular code and implement it in the robot [5]. As for the hardware, the computer specification of AMD Ryzen 5 3600 GPU 6-core processor with 16 GB of RAM and Ubuntu 16.04 is used in this paper to run the software system.

To evaluate the performance of the robot, the estimation map of simulation and real-world experiment is obtained from both algorithms, which is the RBPF algorithm without ANN and with ANN. The map estimation by the robot is then compared to the ground truth map by using the map score function for map evaluation, as mentioned in equation 9:

(9) $$MapScore=1-\left(\frac{1}{N}\sum_{\begin{array}{c} m_{X,Y}\in m\\ n_{X,Y}\in n \end{array}}{\left(m_{X,Y}-n_{X,Y}\right)}^{2}\right)$$

$m_{X,Y}$ is the value of the cell at position (X, Y) in the estimation map; m and $n_{X,Y}$ is the value of the cell at position (X, Y) in the ground truth map; n. *N* is the number of evaluated cells. The closer the value of the map score to 1, the higher the performance of the map estimation. The robot used in both simulation and real-world is a Turtlebot3. To simulate the real robot, which is a Turtlebot3 in Gazebo, a Unified Robot Description Format (URDF) file downloaded from https://github.com/ROBOTIS-GIT/turtlebot3.git is used (accessed on 24 February 2022). URDF file is an XML description of the Turtlebot3 components model and dimensions, kinematic or dynamic model, and sensors attached.

## 3. Results and Discussion

To analyze the performance of the SLAM, the algorithm integrated with the ANN sensor model is compared to the SLAM algorithm without the ANN. There are two phases that were analyzed: first, we validated the model of the ANN after the training using mean-squared error (MSE); second, the SLAM algorithm integrated with the ANN sensor model was compared to the SLAM algorithm without ANN by comparing the map produced by both algorithms using map-score evaluation.

### 3.1. Analysis of the ANN Sensor Model after the Training

After the data collection process, the method described in Section 2.2 was used to train the model by using the Matlab ANN tool named nnstart. In this tool, the Levenberg–Marquardt algorithm, which is an extension of the backpropagation algorithm, was adopted for this network. The training of the model has managed to achieve an MSE value of $9.30\times 10^{-5}$ for simulation and $9.55\times 10^{-5}$ for the real-world experiment.

For the simulation ANN architecture, layer 1 consists of 20 neurons in the hidden layer. For the real-world architecture, 50 neurons in the hidden layer were used. After the training, the synaptic weights in each layer were computed and adjusted according to the lowest mean-squared error (MSE). The training automatically stopped when generalization stopped improving, as indicated by the lowest MSE of the training samples. The weight and bias vectors for this ANN training were determined after several attempts.

**Figure 6:**
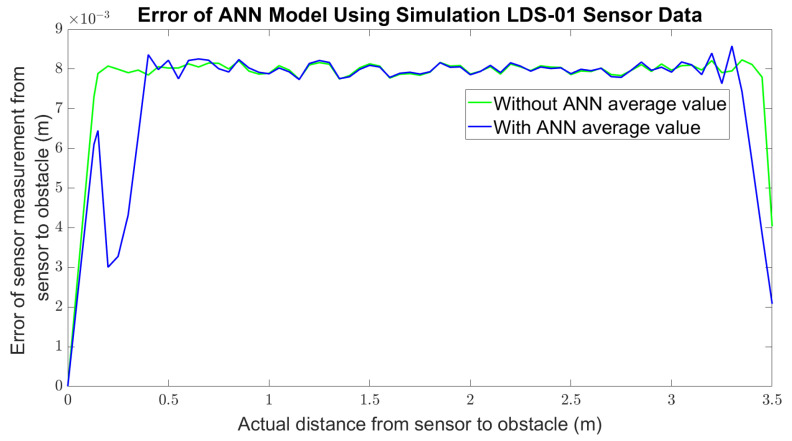
Comparison of error of sensor measurement for simulation.

**Figure 7:**
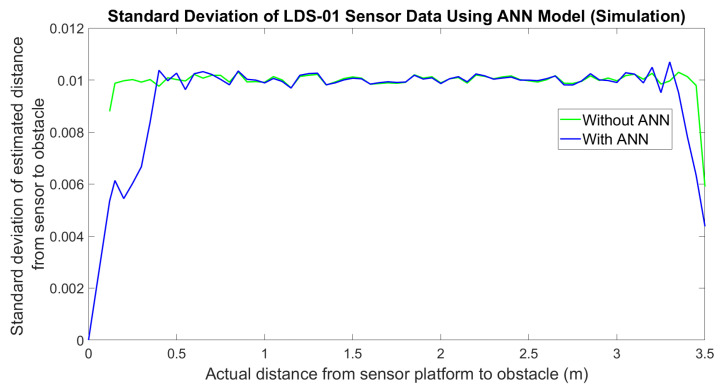
Standard deviation of LDS-01 sensor data for simulation.

**Figure 8:**
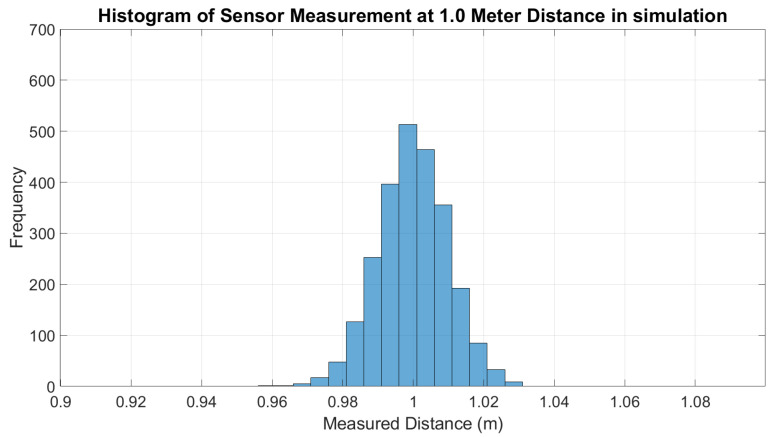
Histogram of LDS sensor measurement at 1 m distance (simulation).

**Figure 9:**
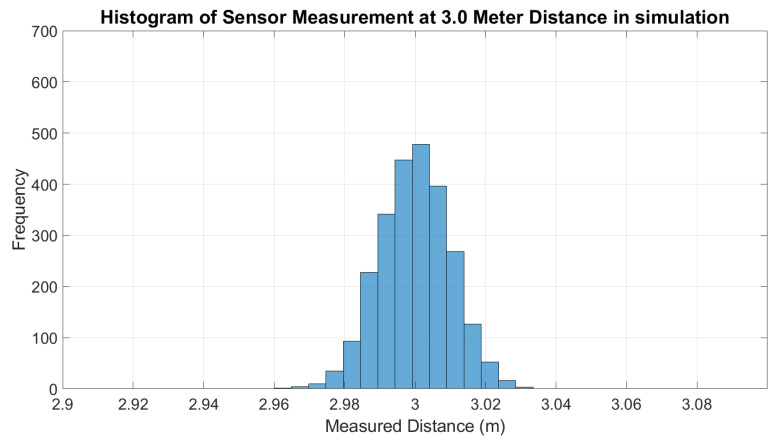
Histogram of LDS sensor measurement at 3 m distance (simulation).

Finally, the ANN model was tested with the LDS sensor measurement data from the simulation and the real world. For the simulation, the error of the LDS sensor measurements without ANN was plotted along with the error of the output of the ANN model applied on each measurement, as shown in Figure 6. The error is calculated by comparing the sensor measurement with the actual distance. From the figure, it can be observed that there is not much difference between ANN and without ANN. Furthermore, the standard deviation of sensor measurements should increase with the distance, as mentioned in [4,37]; however, in the Gazebo simulation, the standard deviation of the sensor measurement in every distance taken is most likely the same, as shown in Figure 7. This is also shown by the histogram of measurements at a 1 m distance in Figure 8 and sensor measurement at a 3 m distance in Figure 9, where both distributions have approximately the same width or standard deviation. It is concluded that there are not many differences in the sensor measurement after the ANN model training. This is because the sensor data that were collected as the ANN’s input have achieved high accuracy of sensor measurement. For each sensor measurement, we collected 2500 sensor data points. The sensor measurement range is around −0.04 to +0.04. When we average the data points, the result is close to the actual distance. For example, in the 1 m distance data collection shown in Figure 9, the data range from 0.96 to 1.04. When we average the data collection of 1 m distance, the result is 0.9999 m, which is very close to the target output of 1 m. As a result, after training the ANN model, the average result of 1 m distance is 1.0001. It shows that the sensor in the simulation has achieved a high accuracy measurement, and the noise of the sensor that has been implemented is less significant. The result is approximately the same across the entire sensor measurement range.

**Figure 10:**
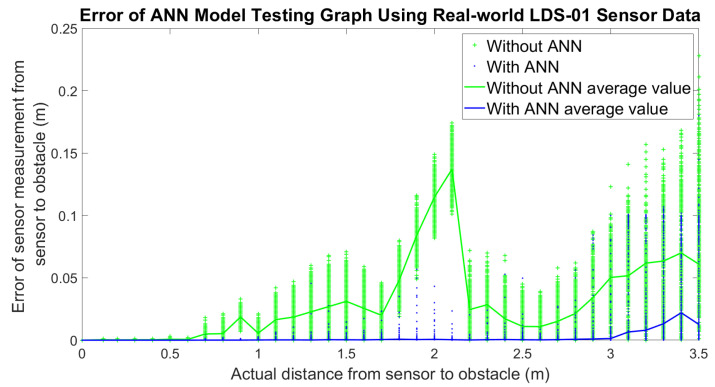
Comparison of sensor measurement for the real world.

**Figure 11:**
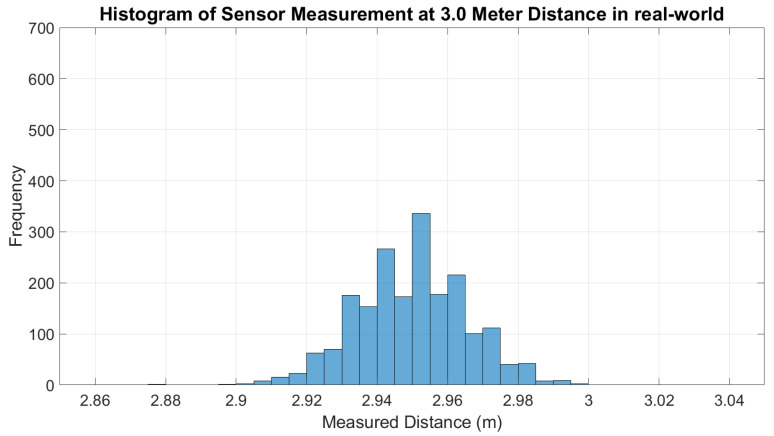
Histogram of LDS sensor measurement at 3 m distance (real world).

**Figure 12:**
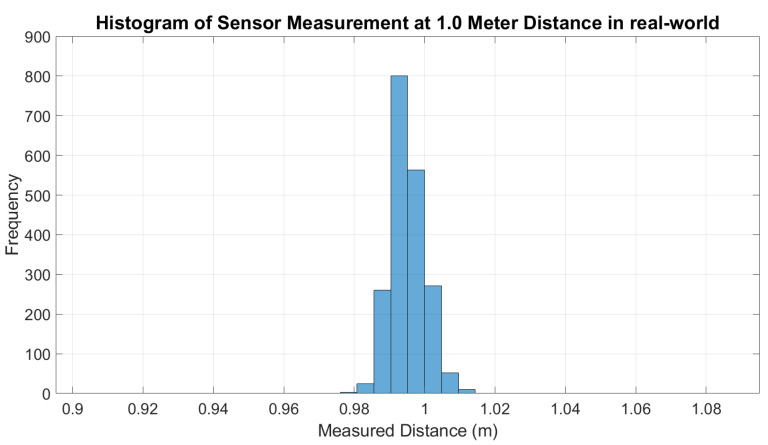
Histogram of LDS sensor measurement at 1 m distance (real world).

**Figure 13:**
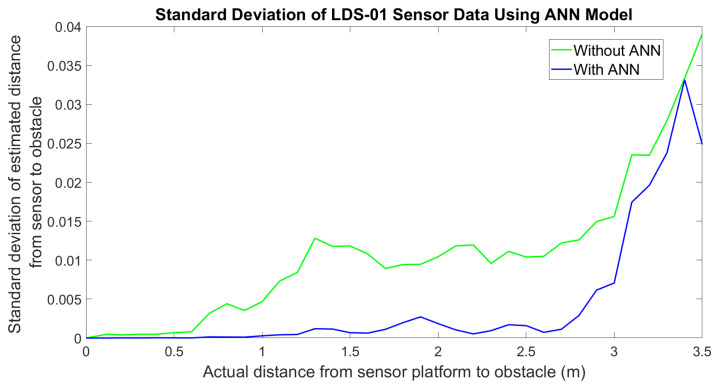
Standard deviation of LDS-01 sensor data for the real world.

For the real-world experiment, the error of the LDS sensor measurement is plotted along with the error of the output of the ANN model applied on each data point, as shown in Figure 10. In the figure, it shows that the error of the sensor measurement before integrating with ANN (green color) is consistently higher. It is also observed that when the range of the distance increase, the distribution of sensor measurements is inconsistent and wider. This shows the noise and non-linearity characteristic of LDS sensor measurements in the reading of the sensor. Figure 11 shows the histogram of the measurements at a 3.1 m distance is wider than the histogram at a 1 m distance, as depicted in Figure 12. Therefore, the standard deviation of the sensor measurements also increases as the distance increase, as shown in Figure 13; however, after the LDS sensor data have been applied to the ANN model that has been trained, the data with ANN have minimal error values compared to the data without ANN, as shown in Figure 10. In addition, the standard deviation of the sensor measurement with ANN (blue color) is lower than the value of the sensor measurement without ANN (green color), as shown in Figure 11. Hence, from these results, it shows that the non-linearity and the error of the sensor readings are significantly reduced by using ANN.

It is necessary for the robot to process data in real time to perform an SLAM task. The proposed method, which is adding the ANN model into the SLAM framework, is only taking $9.54\times 10^{-7}$ seconds to process the sensor data. Hence, the real-time requirement is still met after the proposed algorithm is added—this is because there is no significant cost in computation time.

### 3.2. Experimental Environment

**Figure 14:**
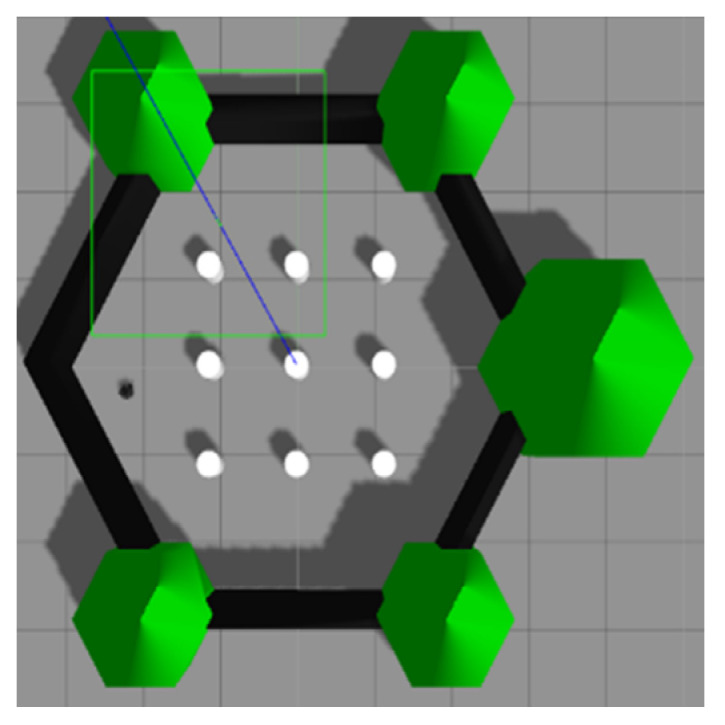
Gazebo simulation environment.

**Figure 15:**
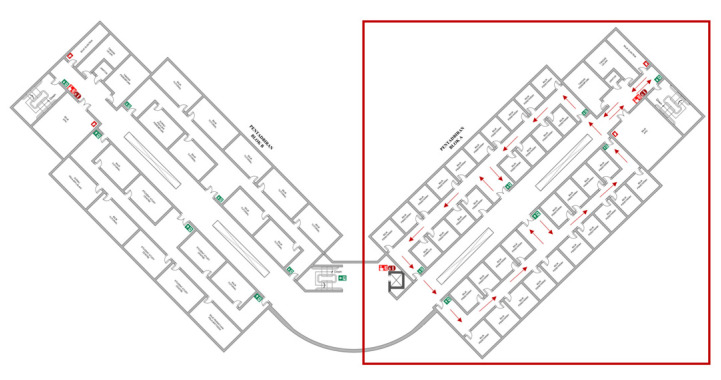
Layout of the real-world environment.

After achieving the desired ANN sensor model that has been validated in Section 3.1, the model is integrated with the RBPF algorithm. Afterwards, this integrated algorithm, as mentioned in Section 2.3, was tested in simulation and real-world environments using Turtlebot3 Burger robot. Primarily, Turtlebot3 is set to navigate in the Gazebo simulation environment, as shown in Figure 14. The size of the simulation environment is 6 × 6 m. For the real-world environment, the experiment was conducted at the Faculty of Electronics and Computer Engineering Universiti Teknikal Malaysia Melaka, as shown in Figure 15. The robot only explored wing A of the faculty (red box) by following the red arrow, as shown in Figure 15. The size of the real-world environment is approximately 43 × 16 m.

In both experiments, the simulation and the real-world environment, Turtlebot3 navigated using the teleop operation. The average speed of the robot was about 0.12 m/s and was simultaneously recorded by using the Rosbag tool. Rosbag is a tool provided in robot operating system (ROS). It allows us to record and playback the data of the robot that have been recorded. Hence, the recorded data can be applied to different algorithms with multiple trials. This way, the RBPF algorithm integrated with the ANN sensor model and without the ANN can be evaluated in equal conditions. Then, the proposed algorithms can be observed for any improvement in accuracy. The data were tested 10 times and evaluated using a map-score evaluation to ensure consistent results. Hence, the results obtained are 10 maps for RBPF with the ANN sensor model and 10 maps for RBPF without the ANN. The results are reported in Section 3.3. 

### 3.3. RBPF Integrated with ANN Sensor Model

**Figure 16:**
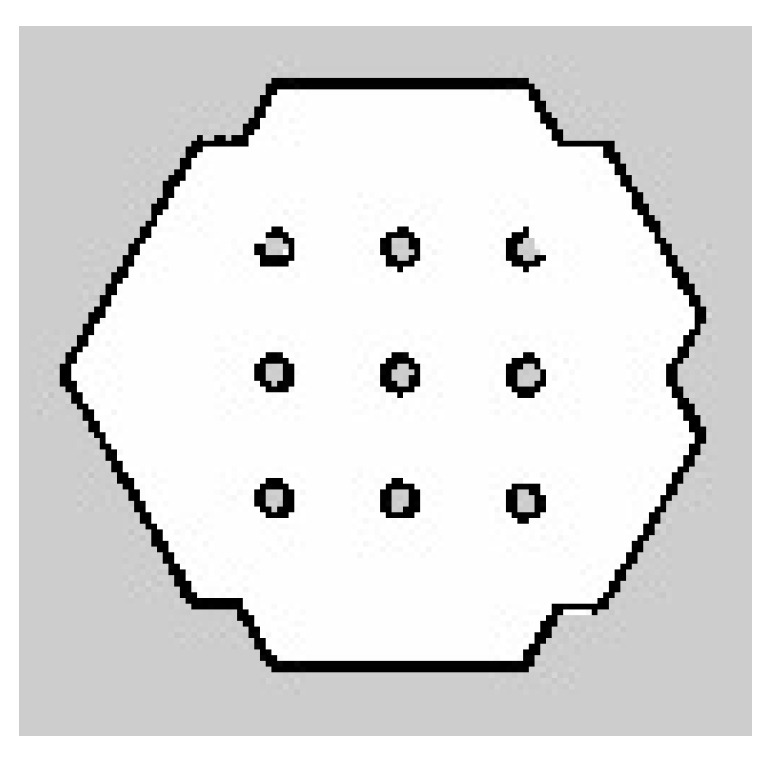
Estimated simulation map without integration with ANN.

**Figure 17:**
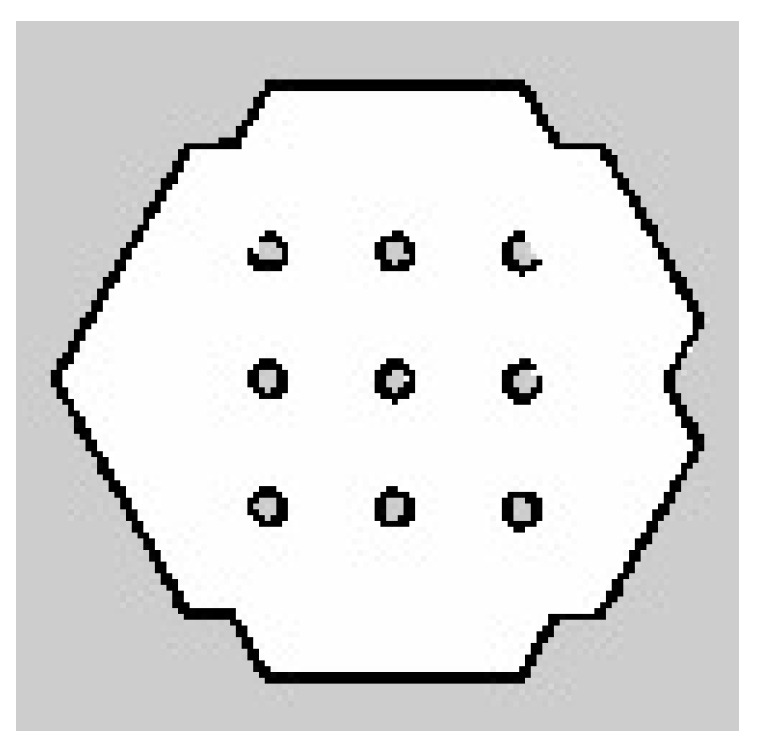
Estimated simulation map integrated with ANN sensor model.

The maps are obtained in the Occupancy Grid map (OGM) representation and saved in pgm format using map saver from the map server package. The size of each grid cell of the map is set to 5 ${\mathrm{cm}}^{2}$. In OGM, the black cells are considered occupied, white cells are considered free cells, and grey cells are the unknown region that has not been explored yet, as shown in Figure 16. Firstly, for the simulation part, the map shown in Figure 16 and Figure 17 is one of the examples of the 10 maps obtained for RBPF without the ANN and RBPF integrated with the ANN sensor model, respectively. Both sets of maps, respectively, are compared with the ground truth map. Visually, there is no significant difference between the resulting maps and the ground truth map.

Then, the maps were evaluated with the map-score function. The estimated map integrated with the ANN sensor model shows a slightly better performance than the estimated map without ANN. RBPF algorithm without ANN has achieved a 0.9915 overall map-score value, while RBPF integrated with ANN managed to achieve a better average map-score value, which is 0.9924—see Table 1. The performance of the overall cells, free cells, and occupied cells based on the result in Table 1, estimated maps integrated with ANN are increased by 0.088%, 0.045%, and 0.498%, respectively, compared to estimated maps without ANN. The improvement of the percentage of map score shows that the RBPF algorithm integrated with the ANN sensor model increases the performance of SLAM a little bit in this experiment.

**Figure 18:**
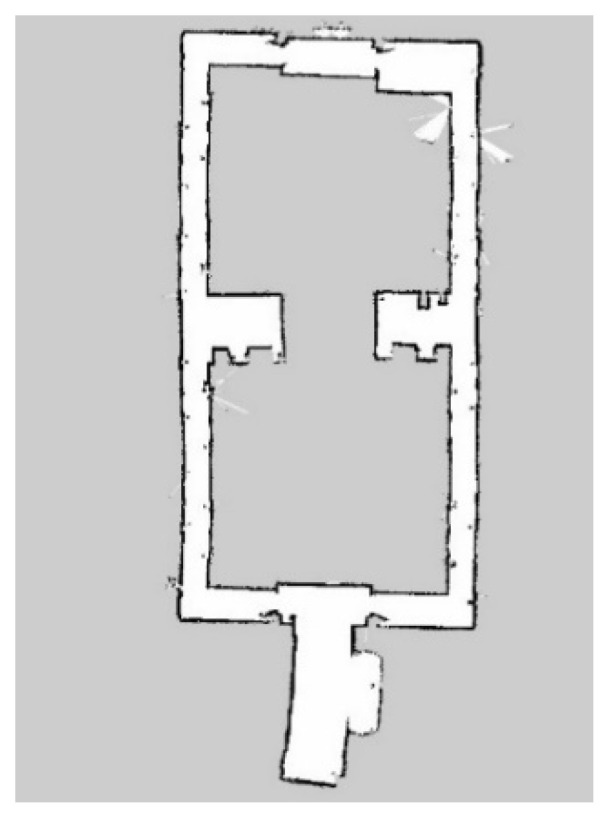
Ground truth map.

For the real-world environment, the resulting maps, which are 10 maps for RBPF with ANN sensor model and 10 maps for RBPF without ANN, are compared with the ground truth map, as shown in Figure 18. The dimension of the map is 1056 × 608 cells. Since the size of the grid cell is 5${cm}^{2}$, this makes the size of the real environment in Figure 18 52.8 m × 30.4 m. Within this map, the explored area by Turtlebot3 mobile robot is approximately 43 m × 16 m, as stated in Section 3.2. 

**Figure 19:**
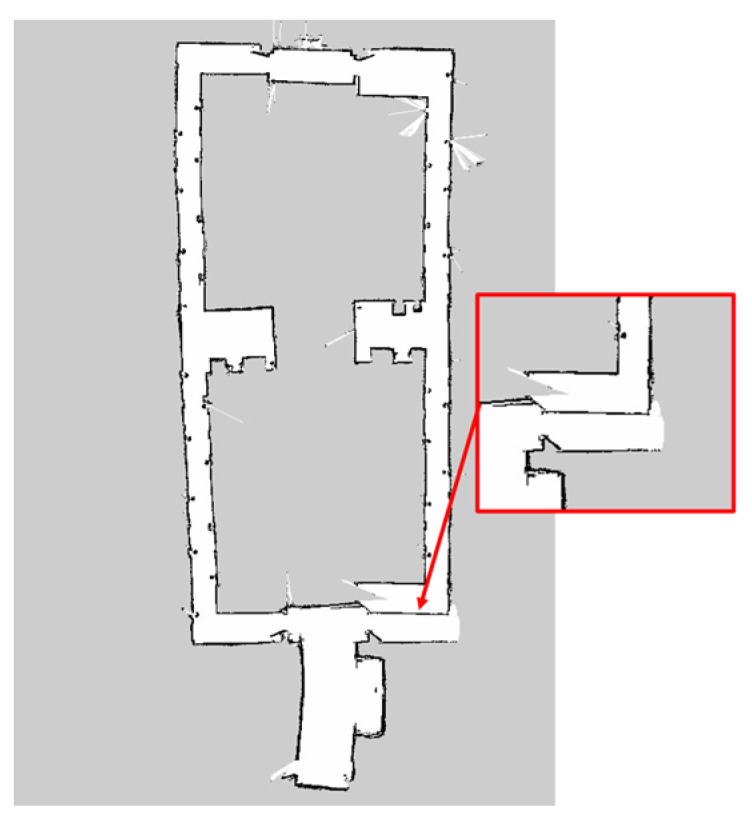
Non-closed loop map without ANN.

**Figure 20:**
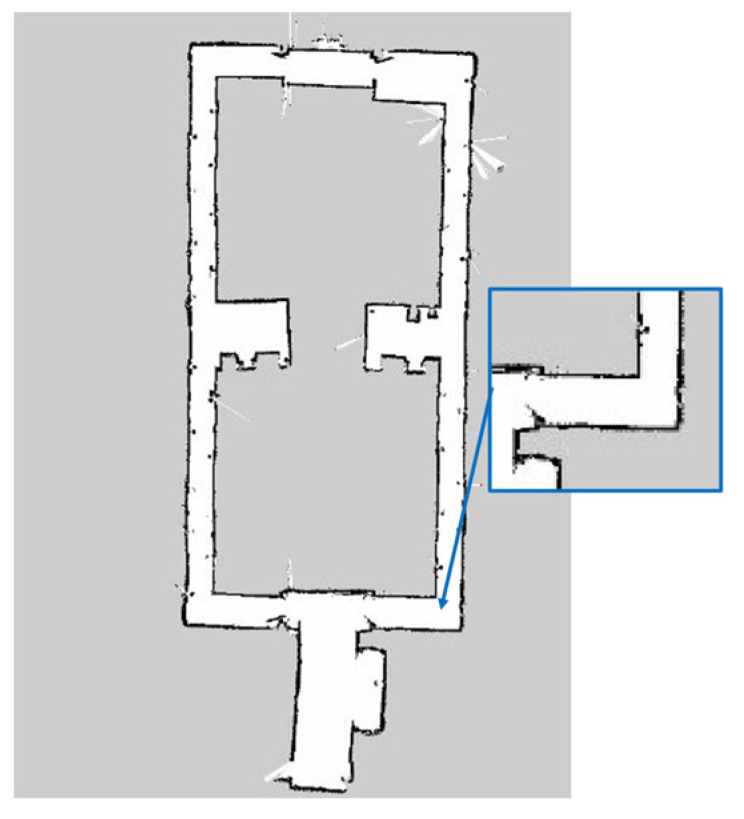
Closed loop map with ANN sensor model.

From the results, it is observed that the RBPF algorithm integrated with the ANN sensor model has achieved all closed-loop map conditions, which is 10 times out of 10 trials. The RBPF algorithm that is not integrated with the ANN does not achieved the closed-loop condition out of 10 trials. The loop closure criteria are one of the main topics in SLAM research. It is defined as the ability to correctly map the revisited area and connect the map into one closed-loop map. Among the resulting maps of the RBPF algorithm without ANN, the algorithm cannot achieve the closed-loop condition that has been zoomed in (red box), as shown in Figure 19. This is due to the low accuracy of the LDS sensor measurement reading that affects the RBPF algorithm; however, with a better reading of the sensor measurement, the reading of the correct environment and closed loop condition (blue box) can be achieved more consistently with ANN as shown in Figure 20. This is because RBPF relies on sensor measurement accuracy to improve the likelihood of its observation. With a better likelihood of observation, the robot’s pose can be estimated accurately, resulting in a closed-loop map condition.

RBPF algorithm without ANN has achieved 0.7929 overall map-score value while RBPF integrated with ANN sensor model managed to achieve a better average map-score value, which is 0.8390 based on Table 2. Furthermore, for the performance of the overall cells, free cells, and occupied cells based on the result in Table 2, estimated maps integrated with ANN have increased by 5.81%, 1.82%, and 107.59%, respectively. The occupied cells’ performance has shown a significant improvement as the map score increased more than twice by integrating RBPF with the ANN sensor model. The accuracy of occupied cells is important because it is used in the RBPF algorithm to determine the weight of particles. In conclusion, the improvement of the percentage of the map score and closed loop condition shows that the RBPF algorithm is better with the integration with the ANN sensor model in this real-world experiment.

## 4. Conclusions

An investigation has been made on the effects of a neural network on the low-end sensor measurements and hence, the SLAM performance. The performance of the SLAM integrated with the ANN sensor model is measured based on the accuracy of map estimates through simulation and real-world experiments. This map is then compared with the map estimated by the SLAM algorithm without ANN to validate if there is any improvement by using ANN to the SLAM algorithm. Both the results in simulation and real-world experiments show that the performance of the SLAM algorithm integrated with the ANN sensor model is better than the SLAM algorithm without ANN. In the real-world experiment results, the performance of the occupied cells integrated with the ANN sensor model has increased by 107.59%. Furthermore, in the real-world experiment, the SLAM algorithm with the ANN sensor model achieved all closed-loop conditions of the map estimate, which is 10 times out of 10 trials compared to the SLAM algorithm without the ANN that does not achieved closed-loop conditions out of the 10 trials. The improvement of the percentage of the map score and closed-loop condition demonstrates that even by using just a low-end sensor, the SLAM algorithm can achieve better accuracy of the map estimation with the integration of neural networks in the simulation and real-world environment. In conclusion, the improvement of the SLAM algorithm using Turtlebot3, which only used low-end sensors as the robot’s perception, is accomplished by integrating the algorithm with ANN.

## Figures and Tables

**Table 1:** The simulation average map-score evaluation for RBPF algorithm without and with ANN sensor model.

	Overall Cells	Free Cells	Occupied Cells
Without ANN	0.9915	0.9959	0.9522
With ANN	0.9924	0.9964	0.9569

**Table 2:** The real-world average map-score evaluation for RBPF algorithm without and with ANN.

	Overall Cells	Free Cells	Occupied Cells
Without ANN	0.7929	0.8912	0.1661
With ANN	0.8390	0.9074	0.3448

## Data Availability

The datasets generated during the current study are available at the github repository, https://github.com/norhidayahm/FKEKK, accessed on 30 December 2022, UTeM.
